# A co-created randomised controlled feasibility trial of a peer-led complex oral health intervention in UK secondary schools: the RAISED In Yorkshire RiY study protocol

**DOI:** 10.1186/s40814-026-01804-y

**Published:** 2026-03-31

**Authors:** Yasmen Elsadek, Aruche-A-Noor Hamid, Poonam Anand, Lucy Simpson, Holly Davis, Flavian Kow, Neha Mehta, Nina Fraser, Abigail Turner, Minnie Lyons-Coleman, David Cooper, SMILE AIDER Patient Public Involvement, Engagement Forum, Sue H. Pavitt

**Affiliations:** 1 School of Dentistry, University of Leeds, Worsley Building Level 6, Leeds, LS2 9LU UK; 2 Dixons Sixth Form Academy, Douglas Mill, Bowling Old Lane, Bradford, BD5 7JR UK

**Keywords:** Caries prevention, Behaviour change, Peer-led intervention, School involvement, Career aspiration, Health promotion

## Abstract

**Background:**

Tooth decay in secondary school children is one of the most prevalent non-communicable diseases in the UK and globally. Whilst almost entirely preventable, oral health inequalities remain a significant public health problem to reach and manage those most vulnerable. School-based prevention strategies targeted to localities with oral health inequality, and incorporating peer-to-peer interventions warrant further evaluation. These peer-to-peer interventions have a bidirectional benefit and are proposed to positively influence both the ‘peer leader’ and ‘peer recipient’; they rely on shared cultural backgrounds and relatability to reach students that may be missed by typical healthcare professional-led interventions. This protocol describes a feasibility trial which aims to establish RAISED in Yorkshire with its peer-to-peer delivery model within the secondary school setting supported by newly qualified dentists as an effective and sustainable approach to improve oral health awareness, skills (toothbrushing) and behaviours in adolescents from underserved at-risk communities.

**Methods:**

This is a UK school-based, assessor-blinded, parallel two-arm randomised controlled feasibility trial with a preliminary interim assessment in advance of progression to a larger scale trial in the future. The trial aims to recruit *n* = ~240 11–13-year-old participants and *n* = ~50 16–18-year-old peer leaders across five schools/colleges in West Yorkshire. The primary outcomes are related to feasibility in terms of recruitment and retention of participants, and the ability to deliver the intervention. The secondary outcomes include the oral health of participants (knowledge, attitudes and behaviour, plaque and gingival index) and process evaluation.

**Discussion:**

The findings of the feasibility trial will dictate whether the project is scalable and can be delivered to multiple schools regionally and nationally. The findings will also show whether it is an effective method to provide oral health education for 11–13-year-old adolescents when delivered collaboratively by trained 16–18-year-old peer leaders.

**Trial registration:**

ISRCTN registry, ISRCTN83511912. Registered on 8 August 2024, https://www.isrctn.com/ISRCTN83511912.

**Supplementary Information:**

The online version contains supplementary material available at 10.1186/s40814-026-01804-y.

## Background

Whilst largely preventable, untreated tooth decay affects more than 3 billion people worldwide [1]. Results of the most recent Oral Health Survey of year 6 children in England demonstrated that approximately one-fifth of children start secondary school with an average of two teeth having tooth decay [2]. The report showed that the children from the most deprived regions of England were twice as likely to experience tooth decay than those from the least deprived regions of the country [2]. Additionally, the UK Children’s Dental Health survey demonstrated an increasing percentage of tooth decay with age; reporting that 43% of 12-year-olds suffered tooth decay followed by 46% of 15-year-olds [3]. Tooth decay in children presents a considerable health and social burden [2–4]. Research involving 11–13-year-old schoolchildren from deprived areas across the UK found that 34.7% had tooth decay in their permanent teeth, with about half of them reporting that their oral health impacted on their day-to-day activities [5]. Additionally, the Children’s Dental Health Survey data showed that 58% of 12-year-olds and 45% of 15-year-olds reported the impact of their tooth and mouth problems on their daily lives [3, 6]. This impact is found to be associated with sociodemographic background, affecting not only the children themselves in terms of poor school attendance and performance but consequently leading to wider impacts on their parents, family and society as a whole [7, 8]. Furthermore, tooth decay constitutes a serious financial burden on the National Health Service in the UK, in 2015–2016 £50.5 million was spent on hospital tooth extractions for children aged 0–19 years [4]. 

Due to the impact of decay on children at schools and the World Health Organisation Assembly’s reinforcement of the importance of a preventative approach to oral health instead of a traditional curative approach, school-based interventions are being implemented with the aim of reducing the burden on individuals and healthcare systems [9, 10]. The success and sustainability of school-based interventions are affected by a myriad of factors, such as school capacity, staff motivation and commitments, duration of intervention, and funding [11]. Although it has been demonstrated that within the school environment, educational outcomes take precedence over health promotion, it has been emphasised that peer interventions remain less expensive requiring fewer resources and yielding healthcare cost savings [12–14]. 

Peer leadership is commonly used in school settings and community groups to influence change and there is growing evidence of effectiveness in terms of health-related knowledge, attitudes, and behaviour change [13]. Peer leadership is when individuals influence and guide their peers by acting as a role model, mentor, and motivator to foster collaboration, trust, and increasingly health improvement. It is built on a foundation of shared cultural experiences and empathy, often adopting a supportive ‘show and tell’ approach, rather than what might be perceived as a more authoritative approach from teacher-led or health-care professional-led initiatives. A recent global systematic review of school-based peer education interventions to improve health highlighted that ‘student-led’ or ‘peer-led’ strategies for health promotion are increasingly common and demonstrate evidence of effectiveness in terms of health-related knowledge, attitudes, and behaviour [13]. This approach has been used to target a variety of public health challenges, such as smoking and alcohol use, mental health, and physical activity [15–17]. 

Peer-to-peer interventions have been reported to be as, if not more, effective in improving knowledge and show more potential in changing behaviour than teacher- or health professional-led interventions [18, 19]. Moreover, other studies evaluated peer-led health interventions and reported positive feedback, including the fun nature of peer-led delivery, the relatability to the peer leader, the credibility of peers, and perceived role model status [20, 21].

RAISED in Yorkshire started through a collaboration of University of Leeds School of Dentistry with a local girls’ high school in an underserved area of ethnic diversity and social deprivation in Yorkshire [22]. Students from this school got involved in research in two ways: (1) Researchers at the university trained 16–18-year-old students to co-deliver oral health education to children aged 7–8 years in local primary schools [23], (2) peer leaders assessed as competent oral health educators conducted oral health research projects as part of their Extended Project Qualification to enhance their career prospects [24]. The majority of these students were ‘first-in-family students’, i.e. first in their immediate family to attend a higher education institution [25]. An unexpected impact of mentoring RiY-peer leaders was raising aspirations and career prospects measured by the first successful applicant to study dentistry [4]. Despite the effectiveness of this co-delivered OH education intervention at improving oral hygiene knowledge, skills and reach to vulnerable communities [23], this model was no longer possible due to the Covid-19 pandemic. Moreover, same-school interventions with closer age gaps were justified in the literature as potentially economical and more available and consequently more sustainable [26, 27]. Existing literature has cited transportation and additional cost for training and delivery at different schools as a barrier to successful and sustainable implementation [18, 26]. Additionally, the oral health of children starting secondary school is often neglected. This led to the shift to targeting 11–13-year-old students in the new model of RiY which would be more of a ‘Lay Health Advisor model’ as they share more characteristics and have more relatability with the 16–18-year-old students.

A recent umbrella review of the effectiveness of oral health education highlighted that oral health education intervention tends to result in only short-term improvement, especially when involving a single one-off session [28]. Hence, evidence-based literature suggests that identifying adult champions and having a reinforcement component of oral health education and promotion play an important role in the sustainability of the effect and increases the likelihood that a newly learned behaviour will be repeated in future [29, 30]. 

Finally, student-led peer-to-peer interventions can result in a bidirectional benefit for both peer recipients and peer leaders, improving oral health awareness, their self-confidence, aspiration, and motivations for the future [31, 32]. A recent systematic review recommends process evaluation to further explore this bidirectional gain and impact [19].

The aim of this trial is to establish if RiY, with its peer-to-peer delivery model within the secondary school setting, supported by newly qualified dentists (NQD), is feasible, effective and a sustainable approach to improve oral health awareness, skills (toothbrushing) and behaviours in adolescents from underserved at-risk communities. Sustainability will be explored from utilising NQD (also referred to as Dental Foundation Trainees) who in England complete a mandatory 1-year post-qualification training programme to practise in the National Health Service (NHS). In that period, they have designated time allocated to volunteer in research and OH prevention projects, like RiY. RiY is the prototype programme under evaluation in this feasibility trial and based in Yorkshire, a locality in the North of England that is reported to have one of the most challenging oral health inequalities in the UK [33].

RiY as a peer-led oral health promotion programme addresses three of the National Institute of Health and Care Research (NIHR) ‘Areas of Research Interest’, notably, early action to prevent poor health outcomes; reduction of compound pressures on the NHS; and shaping and supporting health and social care workforce of the futures [34]. The work aligns with the NHS 10 Year Plan, ‘Fit for the Future’ [35]; RiY addresses two of the three major shifts: moving care from hospitals to community settings, and shifting focus from treating illness to preventing it. Tooth decay is almost entirely preventable yet research involving children aged 11–12 years from deprived areas in the North of England, Scotland and Wales showed over one-third had tooth decay in their permanent teeth and four in ten reported that their oral health had an impact on their daily lives, including pain, eating discomfort, low self-esteem and absenteeism from school [3]. Yorkshire and the Humber compared to England has the highest decay-related hospital admissions for tooth extraction episodes (454 compared to 229 per 100,000 population of 0- to 19-year-olds) with a cost to the NHS of £45.8 million in 2024 [36]. The decay-related tooth extraction rate for children and young people is 3.5 times higher in those living in the most deprived versus most affluent communities [36]. Many interventions have focused on early years and primary school children, reaching and engaging with at-risk adolescents from these underserved communities remains a significant health challenge. This RiY feasibility trial addresses this challenge with a novel co-created peer-led oral health educational intervention that empowers young people to share their newly acquired knowledge and oral health prevention skills towards bringing about behaviour change in adolescent students within their school communities.

## Methods/design

### Trial aims and objectives

The aim of this feasibility trial is to establish RiY with its peer-to-peer delivery model within the secondary school setting supported by newly qualified dentists (NQD) as a feasible, effective and sustainable approach to improve oral health awareness, skills (toothbrushing) and behaviours in adolescents from underserved at-risk communities.

### Trial feasibility questions

1. Is it operationally feasible to deliver Raised in Yorkshire (RiY) to multiple secondary school within the school timetable supported by NQD?
2. Is RiY an effective way to provide oral health education and reinforcement sessions for adolescent/teenagers from years 7 and 8 (11–13-year-olds) when delivered collaboratively by trained peers from years 12 and 13 (16–18-year-olds)?
3. Is it feasible to collect oral (saliva) samples for the ‘YUK - [Yorkshire UK] Oral Bugs’ citizen science project from students participating in RiY? The YUK-Oral Bugs project is co-designed with RiY researchers, peer leaders and teachers. It looks at the impact of toothbrushing on the composition of the oral microbiome.
4. Is it feasible for schools to provide pseudo-anonymised data on RiY participants on (i) free school meal status (proxy for deprivation) and (ii) broad classification on educational attainment.

### Efficacy research question

5. Is there a change in oral health knowledge, skills, and toothbrushing-related oral indices of peer recipients and peer leaders participating pre to post the RiY complex intervention? (To support future trial design).

### Citizen science public engagement research questions (including process evaluation)

6. To assess the acceptability, challenges, and satisfaction of the secondary school partners and key stakeholders (e.g. NQD from Yorkshire and the Humber Deanery) involved in the delivery of RiY in schools.
7. To enable members of the collaboration (e.g. school staff and dental healthcare professionals and NQD) to reflect on their experience of participating in RiY to inform future modifications and capture any benefits/downsides of participation.

### Trial design

The RiY feasibility trial is a UK school-based, assessor-blinded, two-arm randomised controlled trial (RCT). The feasibility trial will be conducted over two to three school academic years with an interim analysis planned at the end of Academic Year 1. The findings of the interim analysis will inform possible changes required to improve operational delivery in subsequent academic years. The study will be carried out in secondary schools in West Yorkshire serving a diverse population with high dental needs. Up to five participating schools will be targeted for this study.

This intervention will be a multicomponent complex intervention comprising two main parts: (1) a short classroom-based oral health education session including three interactive workshops; (2) a reinforcement session including three interactive workshops (YUK-Oral Bugs). Participants in the control group will receive the classroom-based oral health education session after the final follow-up assessments have been completed to avoid contamination of the data collection, but access will be ensured for all to the main RiY session, so that no one is disadvantaged.

#### Study population and setting

A feasibility trial allows identification of key challenges with implementing a complex intervention and operational delivery in the school setting that is unfamiliar with undertaking RCTs. This trial aims to recruit up to five UK secondary schools with an integrated Sixth Form/Sixth Form Colleges and their feeder secondary schools. A formal sample size calculation was not used for this feasibility trial. Rather a pragmatic approach will be adopted informed by the systematic review of Parker et al. [37] looking at health interventions in schools, guidelines developed by Teresi et al. [38] for designing feasibility and pilot studies, and the BRIGHT trial that worked with similar aged adolescent participants delivering an oral health intervention in schools randomised by forms within year groups [5] using a target of *n* = 60 per year group/school. The focus in RiY is on practical considerations, needing to work within a tight budgetary constraint, but considering primarily the number of participants needed to reasonably evaluate feasibility goals of recruitment and retention of students alongside collecting process evaluation data on logistical components of working in school settings located with catchment to an underserved community with high oral health inequalities and often high ethnic diversity, alongside needing to assess the training of peer leaders that is a novel delivery approach of RiY’s oral health promotion. For 11–13-year-old participants, the target recruitment will be at least four classes per school with all students invited (class size is typically *n* = ~30 students/class) achieving *n* = ~240 participants (~*n* = 48 11–13-year-old participants/school); it is expected that this sample size will provide sufficient data to inform a future main trial. This trial will work closely with teachers to ensure that the intervention has minimal interruption to normal school curriculum and timetabling.

For 16–18-year-old peer leaders, the target recruitment will be *n* = ~50 peer leaders (10–12 participants per school/college).

#### Recruitment

Information is provided by Leeds City Council to help identify eligible schools. Expressions of interest will be sought from potential participating high schools via local collaborative connections. The criteria for selecting the schools will be based on having a higher percentage of students on free school meals (FSM) than the national average, used as a marker of socioeconomic deprivation [37]. The school selection will use FSM data published by Ofsted (Office for Standards in Education, Children’s Services and Skills—a government department that inspects and regulates school services in England). Ofsted publishes key school level demographic information, including the percentage of students receiving FSM. In addition, its inspectors use graded judgements to score the quality of various aspects of pupil attainment, behaviours and the schools’ management against a framework for each inspected school. We will use the criteria for selecting schools to incorporate the school ‘Leadership & Management’ organisational standard at good or above as an indicator that the school can manage the coordination activities of being involved in the RAISED in Yorkshire clinical trial.

Email invitations and online meetings will be organised with school headteachers and relevant staff to propose the project and discuss logistical feasibility locally.

#### School eligibility

School inclusion criteria:

- Ofsted rating for ‘Leadership and Management’ rated good and above, an indicator that the school could manage coordination tasks associated with hosting the trial.
- Schools located in or with a catchment from an area of socio-economic deprivation (based on % of children with FSM) supported by Local Authority intelligence and University of Leeds Widening Participation Programme (e.g. Dental Futures).

School exclusion criteria:

- Schools unable to comply with study procedures or schedule

#### Participants

Group 1 Participants—peer recipients:

- Enrolled in a participating UK secondary school
- Students aged between 11 and 13 years at baseline.

Group 2 Participants—peer leaders:

- Enrolled in a participating UK secondary school/Sixth Form College
- Student aged between 16 and 18 years at baseline.
- Exclusion criteria for groups 1 and 2 participants: immunosuppression (for example, undergoing chemotherapy)*
- Severe bleeding disorders (for example, haemophilia)*

*Excluded for participant safety, as the dental examination involves clinical indices ‘bleeding on probing’ that could cause complications in these individuals.

#### Ethical approval

This study has been approved by the University of Leeds Dental School Research Committee (DREC) for both the RiY Main Study (200218/SP/242/A) and RiY YUK-Oral Bugs sub-study (200218/SP/242-B). The principal investigator will ensure ethical approval is sought for any amendments to the protocol or other study documents from the relevant research ethics committee. Amendments to the protocol or other study documents will not be implemented without approval.

#### Development and piloting of the intervention

RiY was co-created with young people aged 16–18 years who were members of SMILE AIDER Patient Public Involvement & Engagement forum, the School of Dentistry, University of Leeds [39]. Key Stakeholder meetings were also held with NHS England Deanery, teachers, and local authorities. Original intervention materials were co-developed by S.P., A.T., and Y.E. in collaboration with DC and other schoolteachers and 16–18-year-old students.

Focus groups were also conducted with young people by YE and SP to aid the co-development of the programme. The intervention was tested in one school in West Yorkshire to assess the practicality, timings, and suitability of materials for the target age group. The procedure of the test intervention is identical to that used in the feasibility trial.

Evidence suggests that interventions tend to be more impactful if delivered and reinforced over time, rather than a one-off didactic session [40]. The RiY intervention is co-designed with young people, for young people, to help create engaging interactive sessions that include hands-on, immersive workshops with further reinforcement workshop sessions, such as the YUK-Oral bugs. All RiY participants will have the opportunity to take part in the YUK citizen science project where all consented students will be engaged in saliva sampling. The plan is to analyse these samples and provide aggregate anonymous feedback of pre and post microbiome composition. The goal is to increase biological understanding of the association of specific harmful bugs and dental decay and improve understanding of the importance of toothbrushing to remove harmful bugs. In summary, RiY provides age-appropriate, curriculum aligned, interactive sessions designed to provide knowledge and skills on the importance of maintaining oral health and reinforcement that covers the science behind tooth decay delivered by RiY trained peer leaders with the ultimate goal to empower young people towards lasting change in oral health behaviour. The expectation is that the RiY intervention will be further refined (following feedback) as a sustainable complex intervention delivered with further fun and informative reinforcement sessions built into subsequent school years should it progress to a full trial.

As part of project planning and co-design, the research team has conducted a series of meetings with young people, stakeholders and advisors to inform the design of a citizen science project; YUK-Oral Bugs was embedded into RiY as an enrichment/reinforcement session. Preliminary results of these meetings and the focus groups conducted with the 16–18-year-old students provided the basis for conducting a demonstrator oral microbiome citizen science project. It was inspired by the citizen science project called *‘Stick Out Your Tongue’* conducted in Barcelona, Spain [41]. RiY includes additional elements, notably a dental examination alongside saliva sample collection, and has pre- and post-intervention data collection. Stakeholders and expert opinions suggested the inclusion of saliva sample collection for oral microbiome analysis may be a useful intermediate measure of oral health change for future trial progression criteria and interim success monitoring as caries change requires a 3-year follow-up. The YUK citizen science project utilises the citizen science model to investigate the potential of creating oral health behaviour change, specifically toothbrushing behaviour and habits, and facilitates a deeper understanding of the change to the oral microbial composition following toothbrushing behaviour change.

Procedure: (Refer to Table 1: The SPIRIT schedule of recruitment, assessment, and intervention delivery).

Consent: (see Additional files 1 and 2).

**Table 1:**
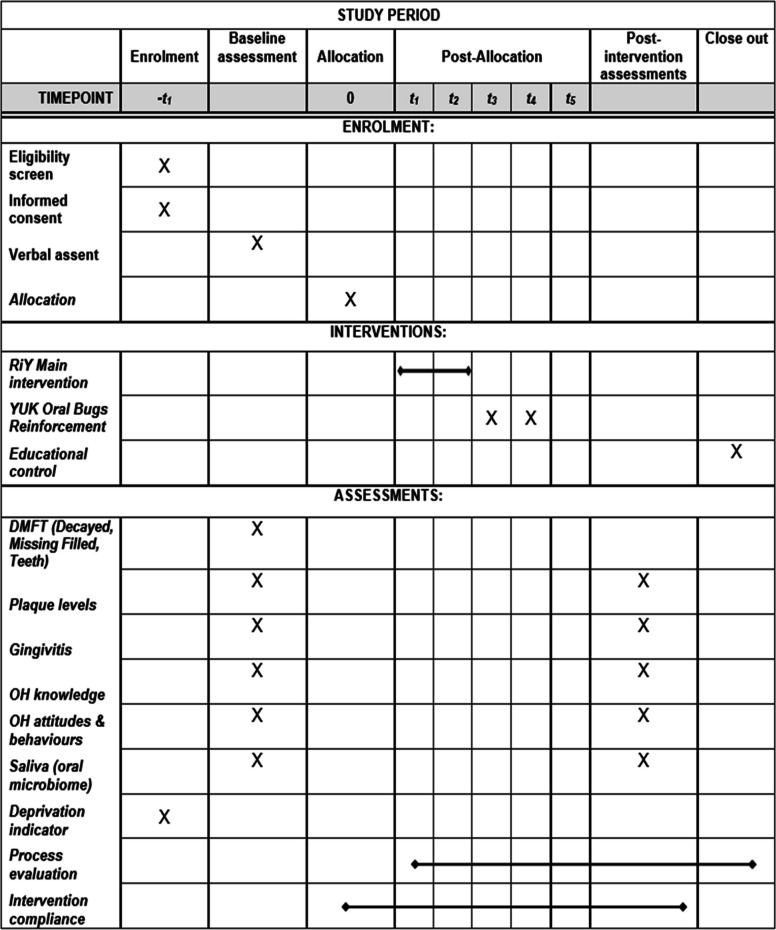
SPIRIT schedule of enrolment, interventions, and assessments

##### Group 1 participants consent

Parental consent is required for participation of the 11–13-year-old students in the RiY trial. Letters will be sent home to parents as per usual school protocol*.* Many secondary schools have methods of secure electronic distribution of letters for student consent, parent/guardian information sheets and consent forms will be distributed through these electronic platforms if this is the preference and standard protocol of the school. The parent/guardian information sheet will explain the purpose of the study and the involvement required and provide an opportunity for parents to agree to participation. The letter will be sent ideally at least 1–2 weeks prior to the sessions to give ample opportunity for parents to provide consent. The consent form will encourage parents to discuss participation with the student to ensure the students and parents/guardians are agreeable and informed. Additionally, an informational video will be produced, reading the parent information sheet in *English and other languages such as Urdu, Arabic, Hindi and Polish*; this will be disseminated to schools to provide increased information and accessibility to parents with English as a second language. To ensure assent from the participants, the research team will deliver a short introductory assembly to ensure the participants are fully informed and happy with the information they have been provided about the programme. They will have the opportunity to ask any questions and refuse to take part without being disadvantaged in any way.

No consent is required to attend the RiY education sessions. Explicit positive consent is a requirement of participation in the programme for collection of oral health indices and questionnaire data. However, the intervention (classroom teaching sessions led by the RiY-peer leaders) will be delivered to the intervention classes; in most schools this will include other non-consenting students. These non-consented students will benefit from the educational component of the teaching but will be omitted from any data collection and analysis.

To facilitate evaluation of the programme and explore added benefits to the RiY-peer leaders, they will be invited to provide written optional consent to undergo dental assessments, saliva sampling (as part of the YUK-Oral Bugs citizen science project), complete oral health questionnaire and quiz. This will allow researchers to evaluate if RiY-peer leaders receive oral health benefits as a result of taking part in the programme. The participants will be fully informed with a detailed Participant Information Sheet and provided with the opportunity to refuse/withdraw without being disadvantaged in any way.

##### Process evaluation

Feedback will be gained from key stakeholders, NQD team and staff at schools predominantly at the end of the trial to explore their experiences/perspectives regarding operational considerations and the opportunity to suggest improvements/modifications to further enhance the RiY programme.

NQD, staff and RiY-peer leaders will be invited to participate in these feedback focus groups or individual semi-structured interview sessions at participants’ preference. NQD can participate as members of the RiY research team without participating in these feedback sessions. The feedback will facilitate the researchers in documenting and evaluating whether the RiY programme can be beneficial for the NQD, peer leaders, and schools and understanding how to improve the programme.

Consent for participation in feedback surveys and/or focus groups or individual interviews will be required from RiY-peer leaders, NQDs and members of staff of the collaborating schools. Participant information sheets will detail the purpose of the feedback sessions, the format of the feedback sessions, the opportunity to refuse participation, anonymisation and storage of collected data. Participant information sheets will be given 1–2 weeks prior to holding feedback sessions, and consent will be collected prior to data collection by the research team.

#### Randomisation

Only Group 1 participants (11–13-year-olds) will be randomised to receive intervention or control on a form level at a 2:1 or 3:1 ratio. As it is a feasibility trial, it will be a convenient proportion based pragmatically on the school’s form structure, timetabling, and availability of our team to oversee it in the individual participating schools.

The size of year group in each participating school will vary, but group allocation will allow for approximately two-thirds of the participants to receive the intervention and the remaining one-third as a control group.

An independent allocator will be given the recruitment numbers per class to perform the randomisation and then inform the schoolteachers of the randomisation allocation.

#### Blinding

Prior to baseline data collection, participants will be assigned individual numerical participant IDs. The assessors (RiY Dentists and NQD, i.e. those undertaking data collection and dental checks) will be blinded to group allocation.

RiY-peer leaders will deliver the intervention to the class and hence cannot be blinded. Nonetheless, they will not participate in data collection. Statisticians and data analysts will also be blinded to allocation. Unblinding procedures are not required for an educational intervention.

#### Intervention

The RiY intervention meets the Medical Research Council definition of a complex intervention in terms of the interactions between components, recognition of the contextual factors and resources required to support reach and impact for implementation and the complexity of oral health behaviours studied [42]. RiY is embedded within the school’s delivery of the national curriculum on dental health and looking after teeth covering the benefits of good hygiene, healthy eating habits, and regular dentist check-ups [43]. The intervention group will receive two oral health education sessions each comprising three interactive, immersive workshops delivered by trained peer leaders assessed as being competent by the RiY research teams as oral health educators using consistent criteria. The *first session* delivers the main RiY intervention that expands on the national curriculum topics and the *second session* is the YUK-Oral Bug reinforcement on the science behind tooth decay introducing the oral microbiome and the importance of toothbrushing. The control group will receive only the standard school curriculum on dental health delivered by schoolteachers. After collection of RiY post intervention follow-up data, the control group will receive a delayed main RiY intervention session, delivered by RiY-peer leaders or teachers depending on timetable availability.

The Group 1 intervention group will attend classroom-based interactive, immersive workshops led by the RiY-peer leaders supported by the RiY Core Research Team or NQD. Each session will be the length of a normal lesson within the school (approximately 55–60 min). This will include three ‘hands-on’ workshops comprising:

1. RiY Main Intervention
  - Sugar (healthy eating)
  - Toothbrushing (oral hygiene)
  - Going to the dentist
2. YUK-Oral Bugs Reinforcement Session:
  - Bugs card game: using flash cards that contain interesting facts about basic oral microbiota (Knowledge)
  - Brushing the germs workshop: using plaque disclosing tablets to demonstrate efficient toothbrushing (toothbrushing skills)
  - Making tooth brushing work for me (strategies for behaviour change)

##### Training of NQD

NQD will be pre-identified by the Associate Postgraduate Dental Dean for Yorkshire and the Humber via expression of interest emails. They will then specify their preference to be involved in teaching, fieldwork, or both. They will then receive the relevant online training for teaching or/and calibration in dental indices.

##### Training of RiY-peer leaders

Expression of Interest will be sought from eligible 16–18-year-old students; the schoolteachers will facilitate the final selection process to the target recruitment of approximately 10–12 students per participating school/college. Training to become a RiY-peer leader includes a 2-day conference at the University of Leeds and an additional YUK-Oral Bugs Reinforcement training session. The selected 16–18-year-old students from the secondary schools/college will attend the conference following written parental permission. The first day will involve didactic teaching introducing RiY, career pathways, *The Science of Tooth Decay* and *Child Oral Health*. The students will then be assigned to groups where each group will practise delivering the three workshops (*Toothbrushing*, *Sugar* and *Going to the Dentist*). They will be trained by NQD and supported by the RiY Research Team.

We will aim for an ~2–3-week interval between days 1 and 2 of the conference to provide sufficient time for RiY-peer leaders to consolidate learning from day 1.

The morning of the second conference day will start with an ice breaker followed by a morning workshop refresher and time to practise in small groups to gain feedback from the supporting NQD and RiY Research team. The afternoon will provide the 16–18-year-old students an opportunity to apply their learning in a real-life situation at the training school located a short coach ride from the University. The NQD and RiY Research Team will support, assess competencies and feed back to the students with the aim to accredit them as RiY-peer leaders competent as oral health educators by the end of the conference.

A further training session for the YUK Oral Bugs reinforcement session will be provided to RiY-peer leaders at their schools prior to delivery of this reinforcement session.

A full risk assessment for the conference day visits to the University of Leeds and training visit to the training school will be submitted to the School of Dentistry, University of Leeds, and shared on request with participating schools.

#### Outcome measures

The following feasibility outcomes have been included to help determine if progression to a full trial is operationally achievable, specifically:

#### Feasibility outcomes (refer to Fig. 1 feasibility study flowchart)

**Fig. 1:**
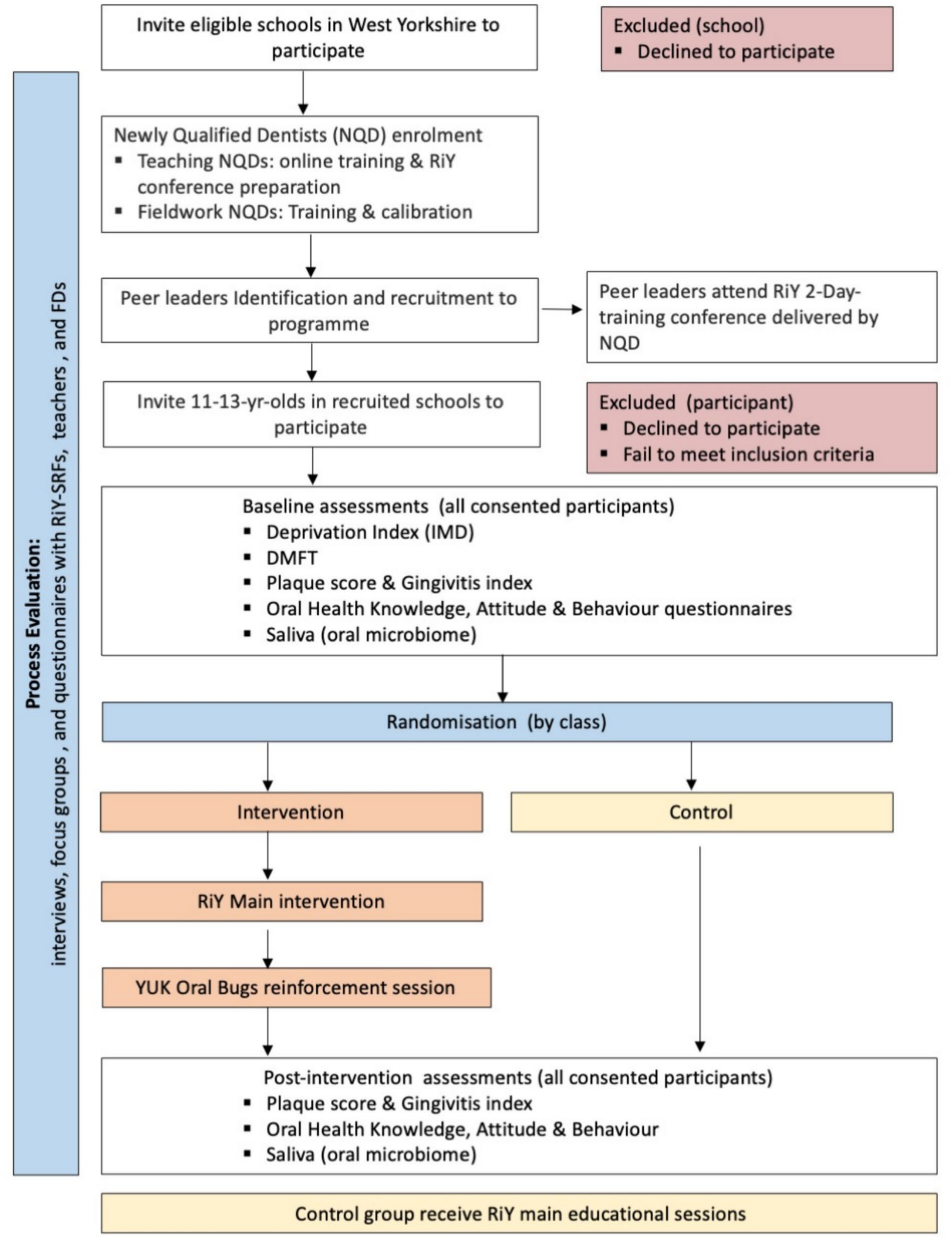
Study flowchart

- Recruitment rate of 11–13-year-old participants, withdrawal, and acceptability of randomisation
- Feasibility of training RiY-peer leaders as peer leader oral health educators
- Feasibility of deploying NQD
- Ability to collect dental health data & link to school's data
- Saliva sample collection for YUK Oral Bugs citizen science project
  I. Number of participants consenting to saliva sampling
  II. Ability to collect saliva samples in schools at baseline and post-intervention

#### Clinical efficacy (secondary outcomes using validated clinical indices and questionnaires)

- Change in toothbrushing skills via Plaque Index using the Turesky Modification of the Quigley-Hein Plaque Index (TQHPI) (calibration of the NQD will be described separately) and Gingival Index using bleeding on probing following the BRIGHT trial protocol for the same age students (baseline and post-intervention) [5, 44]
- Knowledge/attitude/behaviour via Adapted WHO Oral Health questionnaire (baseline & post-intervention)[45]
- DMFT/dmft score (recorded only at baseline)

#### Process evaluation

- Questionnaires developed by the RiY research team based on the topic guides for the focus group/semi-structured interviews are available for use with key stakeholders (e.g. NQD, RiY-peer leaders and teachers) to understand RiY experience, school logistics
- Impact of RiY on school, year 7/year 8 students, logistics and feasibility of student participation.

#### Data collection

Consented participants will be allocated a study ID number that identifies their participating school and unique identifier; this allows for pseudo-anonymisation and confidentiality of data. The data will be collected by suitably trained, qualified and calibrated NQD, supervised by the RiY research team from the University of Leeds. Data Sharing Agreements will be put in place with each participating school/college to allow General Data Protection Regulation (GDPR) compliant transfer of study-relevant data between each school and the University; the data handling procedures will be in accordance with the GDPR Act 2018.

##### Group 1 Participants-peer recipients

The following assessments for student participants aged between 11 and 13 years will be completed at baseline and post-intervention:

- Dental assessment to record DMFT/dmft (at baseline only), Plaque index and Gingival Index. Each participant will brush their teeth allowing assessment of toothbrushing skills; ‘bleeding on probing’ is not influenced by precleaning teeth and is a proxy marker for longer term behavioural change. These are used in the feasibility trial as interim clinical markers of success but in the full trial there will be longer term assessment using the ICDAS (International Caries Detection and Assessment System (ICDAS) scores/DFMT as measure of effect on caries prevention) [46].
- Complete questionnaires:
  o Adapted WHO Oral Health Questionnaire for children [45] (Leeds online surveys/Google forms or paper form according to school preference). It is a widely used and validated tool enabling countries to conduct standardised oral health surveys that are comparable internationally.
  o Quiz about oral health knowledge, attitudes and behaviours
- Saliva sample collection as part of YUK Oral Bugs citizen science project. Consented participants will provide a saliva sample by chewing on paraffin wax and drooling into a tube for approximately 5 min to collect approximately 5 ml.

##### Group 2 RiY-peer leaders

- RiY-peer leaders will be invited to complete an online survey which will take approximately 15 min to complete.
- Participants will be invited to a focus group or interview to explore their experiences of being involved with the RiY programme.
- The RiY-peer leaders will be invited to undergo optional dental exams and assessments and saliva collection (the same as group 1 years 7 and 8 participants). This is to explore the added benefits/impacts of the programme to their own oral health. Data collection will occur at baseline and follow-up in line with Group 1 participants.

##### NQD

- NQD will be invited to take part in feedback sessions to fulfil feasibility outcomes.
- NQD will be invited to complete an online survey. The results of the online survey will facilitate topic guide creation for further focus group discussion.
- NGD will only be considered ‘participants’ to take part in the feedback sessions of the programme. NQD can participate as members of the research team without participating in these feedback sessions. Consent will be required to participate in these feedback sessions.

#### YUK-saliva processing

Tubes of saliva will be labelled, transported, and processed according to DenTCRU, University of Leeds local working instructions for oral microbiome analysis. Participants will not be identifiable on these labels. These will ensure compliance with GOV.UK packaging and transport requirements UN 3373 P650 standard [47].

The saliva samples will be stored in a University of Leeds DenTCRU −80 Freezer or −20 Freezer for RiY processing for shotgun or 16S (commercial) sequencing. 16S sequencing summary compositional oral microbiome reports will be generated and SPSS software or other specialist software will be used for statistical/descriptive analysis of data in addition to the production of heatmaps using the R software package.

Samples will be subjected to shotgun/16S profiling using a commercial platform such as Illumina MiSeq technology or equivalent delivered via an extraction kit or equivalent resource. The data will be available for scientists and processed bioinformatically.

#### YUK biofeedback

A summary of simplified oral microbiome stomatypes will be used for the school biofeedback session. This will be simplified aggregate data that ensures confidentiality and ensures no stigmatisation of any individuals. It aligns with the YUK-Oral Bugs reinforcement session to relay information relating to any changes in bugs related to participants receiving the intervention from their newly acquired knowledge, skills and behaviour of oral health and tooth brushing compared to their baseline sample. Having control group samples will allow identification of any additional influence relating to oral health in the school background.

The feasibility of producing school summary aggregate biofeedback data in a timely manner will be assessed. Stakeholders will be asked to provide comment.

#### Data analysis

The outcome data of this feasibility trial will be reported in accordance with CONSORT 2010 statement extension to randomised pilot and feasibility trials [48].

#### Quantitative analysis

Participant data will be entered electronically on a password protected Microsoft Access database (Trademark of MICROSOFT Corporation, One Microsoft Way, Redmond, WA, 98052-6399, USA) stored securely on the University of Leeds cloud server and accessed via encrypted University of Leeds devices only. Data will be pseudo-anonymised by assigning a unique identification number to each participant. The final data set will reside in DenTCRU, University of Leeds. Data sharing agreements are in place with all schools to allow participant details to be shared and stored. Descriptive analysis will be undertaken by research statisticians using SPSS software. Planned interim analysis will be undertaken to address feasibility outcomes and compare against the progression criteria. The findings will inform possible changes required to improve operational delivery of the trial.

Feasibility, the primary outcome of this study, will be evaluated by calculating the percentage of recruited participants from the target recruitment number of participants overall (recruitment) and the percentage who complete the intervention and have follow-up data (retention).

#### Qualitative analysis

Recordings of semi-structured interviews and focus groups undertaken as part of the evaluation process will be transcribed verbatim. Data familiarisation, coding and interpretation will be conducted independently by two members of the research team to identify relevant themes. Qualitative findings will be utilised alongside quantitative outcomes related to process evaluation to explore how RiY was experienced by participants and any changes to help improve the intervention. The plan is for summary trial results to be shared with the schools.

#### Adverse events and serious events procedures

The study is covered by the University of Leeds Public Liability Insurance.

#### Serious adverse events and adverse events

Adverse events (AE) and serious adverse events (SAE) related to the study intervention will be collected and recorded in the adverse event log.

Participants may experience minor discomfort, becoming upset or may experience minor bleeding as a result of the dental exam. Similarly, during the trial, participants may also experience unrelated health incidents. These events will not be recorded as part of the safety reporting of the intervention. We only seek to record those that could be related and unexpected.

An event is defined as ‘related’ if the event was due to the administration of any research procedure. The relatedness of an event will be reviewed by the principal investigator.

#### Reporting adverse events

Details of any SAEs or AEs will be reported to the PI by the research team. Only details of any SAEs that are required to be reported to the Research Ethics Committee, i.e. SAE events which are related to taking part in the study and are unexpected, and AEs that are related and unexpected will be recorded using a trial adverse event form. The AE reporting period for this trial begins as soon as the participant consents to be in the study and ends at the final data collection point.

#### Reporting urgent safety measures

Safety issues will be reported to the DREC in the annual progress report. An annual summary of all events will also be reported to the Sponsor on request. Expedited reporting of events to the DREC and the Sponsor will be subject to current DenTCRU Standard Operating Procedures (SOPs) and Sponsor requirements.

#### Suspected serious pathology

In rare events where serious dental or oral pathology has been suspected (for example, oral cancer, sepsis or unexplained swelling) and identified during clinical examination, the Chief Investigator or Principal Investigator will discuss with a second colleague in line with good practise, to decide on the most appropriate person for the participant to be referred to. If the common consensus is to refer the participant, the school will be contacted and DenTCRU will work closely with the school and school nurse to ensure the participant receives the help in their best interest and safety.

#### Safeguarding

RiY has strict safeguarding procedures in place that have been ethically approved. In summary, our process is that any concerns identified during the dental checks will be communicated directly to the School safeguarding lead. The school has clear instructions and a detailed flow diagram to follow depending on whether the child has a registered dentist or not. It is the school’s role to liaise with parents/guardians.

## Discussion

This feasibility study is a result of the need for a sustainable method to address poor oral health in secondary school students, reduce oral health inequality in areas of high social deprivation and increase aspiration and STEM (Science, Technology, Engineering and Maths careers) opportunities for 16–18-year-old first-in-family students [25]. Data relating to raising aspirations of the peer leaders will enable the wider impact of participating in the RiY programme to be captured. In these schools, the students often lack parental-initiated opportunities for work experience and university connections to raise aspirations to apply for higher educational continued education. The impact on peer leaders of participating in the RiY Programme will be explored in YE doctoral studies.

The RiY trial is one of the first school-based peer-led interventions targeting oral health behaviour change in adolescents in the UK. It is also a pioneering evaluation of oral microbiome data being collected and analysed from at-risk adolescents. Many oral health prevention programmes exist and are implemented in schools by either teachers or health and social-care workers [49], but RiY is novel in its peer-to-peer approach and the benefits proposed are likely to affect participants and support NHS workforce development by engagement of the NQD in leadership and research opportunities.

A strength of this feasibility study is the recruitment of several local schools across the local area as it improves the generalisability of the findings in the West Yorkshire region. The authors will work closely with participating schools to ensure that the intervention can be incorporated into the curriculum with minimal disruption to the timetable.

The use of NQD could be seen as a limitation of the study data collection. However, great lengths have been taken to train, calibrate and up-skill them to be competent assessors. They are working completely within their professional scope; plaque scoring and bleeding on probing are all commonly used clinical indices. If RiY progresses to a full trial, follow-up would require more complex ICDAS scoring and full consideration would be given to having trained and calibrated clinicians for ICDAS scoring to ensure the highest standards of data integrity. Nonetheless, NQD offer a fantastic sustainable approach to deliver school-based dental public health research. The benefits to these NQD are being explored further in a separate study by NF and NH.

The findings of this trial will inform the design of a future definitive randomised controlled trial if it is shown to be feasible. The RiY concept is expandable from Yorkshire to other geographical areas as ‘RAISED in … *[insert new locality]*’ to provide local ownership, relatability, and pride in the programme, supported by NQD in these localities could offer a sustainable approach that warrants further health economic evaluation. The current feasibility protocol is focused towards advancing mechanistic understanding with the inclusion of YUK. Preliminary health economic assessments are not possible within this RiY feasibility trial due to current funding constraints but this will be important to include in the definitive trial. At an upstream level, this could be adopted to improve oral health prevention policies and guidance to reach at-risk adolescents in areas of high oral health inequality.

Progression to main trial will be based mainly on recruitment and retention, but with other considerations that may influence modifications or remedial action, if needed prior to progression (Table 2). Facilitators and barriers to recruitment and data collection will be identified and strategies will be refined and tested in subsequent year 2 (3 if required) to further inform the main trial.

**Table 2:** Progression criteria to progress to full trial

	**Automatic progression**	*Progression with modification if appropriate*	*Remedial action with key stakeholders/advisory group*	
**Recruitment** % of target number of 11–13-year-old participants recruited (~240 participants (~*n* = 48/school)	** > 75%**	> *50–*< *75%*	< *50%*	
Recruitment of schools *n* = 4 (numbers enrolled)	** > 75%**	> *50–*< *75%*	< *50%*	
**Retention** % of recruited participants with follow up data	** > 65%**	> 40–< 65%	< 40%	
% of recruited schools with follow up data	** > 65%**	> 40–< 65%	< 40%	
**Randomisation:** % of contamination in the control group (whether feasible to randomise by class or by school)	** > 75%**	> *50–*< *75%*	< *50*	
**Other considerations that may influence modifications/remedial actions:**				
• Recruitment and retention of 16–18-year-old students *n* = 10–12/school/sixth form college • Confirmation of feasibility of consent process (analysis of rates for questionnaire/dental assessments and saliva sampling) • Confirmation of operational delivery to intervention and control groups and suitability of embedding educational component within curriculum Confirmation of feasibility to collect clinical data, questionnaires, dental checks and saliva • Assessment of data quality of questionnaire response completion • Confirmation of positive effect on toothbrushing at approx. 10–12 weeks: a. Pre- and post-intervention (individual) b. By randomisation group				

## Supplementary Information

Supplementary Material 1. 

Supplementary Material 2.

## Data Availability

The datasets generated during and/or analysed during the current study are not expected to be made available due to Data Sharing Agreements with schools/college exclusively with the University of Leeds and do not allow access to third parties due to the sensitive nature of data.

## Abbreviations

AE: Adverse events
BOP: Bleeding on probing
DenTCRU: Dental Translational and Clinical Trials Unit
DMFT/deft: Decayed missing filled teeth/decayed extracted filled teeth
DREC: Research Ethics Committee
FSM: Free school meals
NQD: Newly qualified dentists
NHS: National Health Service
PPIE: Patient and Public Involvement and Engagement
RCT: Randomised controlled trial
RiY: RAISED in Yorkshire (Research Activity in Schools Evaluating Dental health)
SAE: Serious adverse events
SMILE AIDER PPIE forum: Stakeholder Meaningful Involvement and Engagement Aiding Dental Research PPIE forum
SOPs: Standard Operating Procedures
STEM: Science, Technology, Engineering and Maths careers
WHO: World Health Organisation
YUK: Yorkshire, United Kingdom Oral Bugs

## Declarations

### The Trial Steering Committee (TSC)

A Feasibility joint TSC (also covering Data Monitoring (and Ethics) Committee DM(E)C.

has been formed and includes an independent chair and other independent members, such as a statistician, clinician and patient public involvement representatives. Sponsor and funder representatives can also attend as observers. Other members of the research team may attend meetings as and when necessary. The TSC will provide overall guidance of the trial and review feasibility outcomes, modifications and progression criteria. They will ensure that the study is conducted according to the protocol and within the overarching ethical framework. Members will also provide advice outside these meetings according to their area of expertise at key stages.

**Table:**

Name	Role
Professor Robert West	Chair, Statistician
Professor Brian Nattress	Independent clinician DenTCRU Clinical Lead
David Cooper	School Educational Lead Advisor
Richard & Jenny Boards	Chair & Member of SMILE AIDER PPIE Forum [39]
