# Supplementary material for: A co-created randomised controlled feasibility trial of a peer-led complex oral health intervention in UK secondary schools: the RAISED In Yorkshire RiY study protocol
Source: Pilot Feasibility Stud. 2026 Mar 31;12:63. doi: 10.1186/s40814-026-01804-y (PMC13162352; doi:10.1186/s40814-026-01804-y)
Supplement: Supplementary file 1 — Supplementary Material 1. [file 40814_2026_1804_MOESM1_ESM.pdf]

RAISED In Yorkshire
RiY RESEARCH ACTIVITY IN SCHOOLS EVALUATING DENTAL HEALTH

1. WHO ARE WE?

Tooth decay (rotten teeth) can have a huge impact on a child's life. As dental professionals, we want to provide resources for children in our local area to improve their oral health. Your child's school has partnered with the University of Leeds, School of Dentistry, to take part in our project: RAISED In Yorkshire (RiY). RiY has been co-designed with young people, teachers, and oral health experts to improve tooth brushing habits. The aim is to empower young people with the skills and knowledge to improve their own oral health habits. RiY is a Citizen Science project that provides opportunity for young people to be engaged in science that is important to them. We will provide valuable education and resources to your child's school, to improve student oral health. Qualified dental professionals from the University of Leeds have been working with keen sixth form students from your child's school as part of the RiY programme. These students have been working hard to become oral health educators and deliver interactive and useful sessions that will raise awareness and knowledge about dental health.

2. WHY ARE WE DOING IT?

Yorkshire & Humber has the worst oral health in 12 years-olds in the UK. Almost half of students start secondary school with rotten teeth. Poor oral health leads to toothache, rotten teeth, bullying, low self-esteem, and time off school. Tooth decay is preventable. Reaching adolescents and teenagers at-risk of oral health inequalities is not easy. In RiY oral health experts will train sixth form students to deliver lessons to Year 7 students to help improve their tooth brushing skills, knowledge, and habits to prevent tooth decay.

3. WHAT WILL THIS INVOLVE?

IN THE CLASSROOM - EDUCATION

Trained sixth form students from will deliver fun and interactive workshops in your child's classroom. This will align well with the school curriculum and will educate the children about how to look after their oral health.

Your child's class will be allocated to one of two random groups. In one group, the children's class will attend a lesson about Oral Health and Hygiene delivered by students from the sixth form. The other group the children's class will receive the teaching later in the year once we have finished the study. This is a common method in research to test if a new programme works compared to the old way of doing things. But once our RiY programme is done, we will ensure that teachers have access to the resources we use so no children miss out.

3-1 ORAL HEALTH KNOWELDGE

Your child will be asked to complete some questions to find out what they already know about looking after their teeth.

3-2 DENTAL HEALTH ASSESSMENT

A team of dental professionals will come to school to perform a dental check-up at two intervals throughout the year. The dental assessment will not replace your child's usual dental check-up. During this check, they will record the health status of your child's teeth and perform a simple check of how clean their mouth is. Your child will be given a toothbrush and asked to clean their teeth. We will look at how good their toothbrushing is. No treatment will be provided, we are only looking at their teeth.

3-3 PHOTOGRAPHY

We would like to take some photographs/videos of the children taking part in the RiY and YUK Project to help us show and promote to other school children, scientists, and dental health professional how we have been working in partnership with your child's school to improve oral health. Above all the School's policy on taking and the use of photos/videos will be adhered to. We seek your consent to take photo's during the activities described here.

4. CONSENT TO PARTICIPATE

We hope these oral health sessions co-designed with young people will be fun and will help your child find science interesting and inspire them to look after their teeth. There will be qualified dental professionals and school staff to supervise all the activities. Your child will not be excluded from the classroom based Oral Health education sessions with the rest of the class, but we would like to make sure you are happy for your child to take part in each activity described in Section 3: notably Section 3-1 surveys, Section 3-2 dental exams, and Section 3-3 photographs. Please complete the consent form sent with this leaflet and return it to the school. Please indicate which activities you are happy for your child to be involved in. We encourage you to discuss participation with your child.

5. WHAT WILL YOU DO WITH MY CHILD'S DATA?

All children in forms enrolled in RiY will receive the classroom based oral health education lesson. Only the children who have returned parental consent forms will be able to take part in the activities described in Section 3 (surveys, dental exam and photographs).

We will collect information from the school about if your child receives free school meals and their broad educational attainment category (e.g. low, medium, high or by streaming group), but this will be pseudo-annonymised to maintain data confidentiality. This data will enable us to assess if the oral health knowledge gain is achieved inclusively and to all ability children. Data will be handled in accordance with the Data Protection Act 2018. The data from the surveys will be kept secure and stored on University of Leeds secure drive which is password protected. The data will be pseudo-anonymised and assigned an unique study ID number.

For more information about how the University of Leeds uses personal data for research, please follow the following web address: https://dataprotection.leeds.ac.uk/research-participant-privacy-notice/

6. WHAT WILL HAPPEN TO THE RESULTS OF THE STUDY?

All the information that we collect about your child during the RIY research will be kept strictly confidential. We will take steps wherever possible to pseudo-anonymise the research data; your child will not be identified in any reports or publications. In the future, we hope to publish the findings in journals and at both national and international meetings. We also intend to provide feedback to the schools as group anonymised data of the findings of the research.

7. ANY QUESTIONS?

We recommend all children see the dentist for regular check-ups. If your child is not registered with a dentist you can contact NHS Direct on 0300 311 2233. They will give you a list of NHS dentists in your area.

If you have any concerns about your child's teeth you need to speak to your dentist. The dental team visiting cannot provide advice to children about the health of their teeth. If you have any questions about the school visits you can contact the dental team on the email overleaf. They can tell you more about the visits, but they cannot give you dental health advice. Staff at your child's school may also be able to answer any questions you may have.

CONTACT

Professor Sue Pavitt (Director of the Dental Translation and Clinical Research Unit, University of Leeds)
Email: dentcru@leeds.ac.uk
Yasmen Elsadek (Dental & Hygiene Therapist, University of Leeds)
Email: dnyeee@leeds.ac.uk
