# Supplementary material for: A co-created randomised controlled feasibility trial of a peer-led complex oral health intervention in UK secondary schools: the RAISED In Yorkshire RiY study protocol
Source: Pilot Feasibility Stud. 2026 Mar 31;12:63. doi: 10.1186/s40814-026-01804-y (PMC13162352; doi:10.1186/s40814-026-01804-y)
Supplement: Supplementary file 2 — Supplementary Material 2. [file 40814_2026_1804_MOESM2_ESM.pdf]

PARENT/GUARDIAN CONSENT FORM for RAISED IN YORKSHIRE and 'YUK -Oral Bugs' Project

<<Insert School name >>

Full Name (Child) ………………………………………………Class ……………………………………………..

Please only add your <u>initials</u> to the boxes where you agree with the statement

Statement	Initials
I acknowledge that I have read and understood all the information in the parent/ guardian information sheet provided. I have had the opportunity to consider the information and ask questions if needed	☐
I understand that I have a choice over which activities decribed in Section 3 of the parental information sheet I agree to provide consent for my child to take part in.	☐

3-1 CLASS ROOM SESSION Oral health Knowledge Surveys

Statement	Initials
I give consent for my child to complete the oral health questions as part of the oral health education sessions	☐

3-2. DENTAL ASSESSMENT

Statement	Initials
I acknowledge that I have read and understood all the information in the parent/ guardian information sheet provided about the dental assessments.	☐
I confirm that my child is not immunosuppressed and does not have any severe bleeding conditions (e.g., haemophilia)	☐
I give consent for my child to have dental assessments at school.	☐

3-3 SALIVA SAMPLING – YUK Oral Bugs Project

Statement	Initials
I acknowledge that I have read and understood all the information regarding saliva (spit) sampling in the parent/guardian information sheet.	☐
I understand that the saliva sample will not have my child's name on it; it will have a unique study ID number with the code securely stored separately.	☐
I give consent to collect a saliva sample from my child	☐
I give consent for my child's saliva sample to be stored securely in the School of Dentistry University of Leeds designated freezer for up to 1 year post the end date of the RiY Programme (30/09/2029) and understand it may be used for future research for the RiY project. After this time, the sample will be destroyed following appropriate Standard Operating procedure.	☐

Please complete and return this form by <<(DD/MM/YYYY)>>

Please only add your <u>initials</u> to the boxes where you agree with the statement

3-4 PHOTOGRAPHY

I acknowledge that I have read and understood all the information regarding taking photographs/video in the parent/guardian information sheet. ☐

I understand that may photographs/video will follow my child's School's policy on taking and the use of photos/videos. ☐

I consent to the RiY University of Leeds team taking photographs that include my child to promote to other school children, scientists, and dental health professional how they have been working in partnership with my child's school to improve oral health. ☐

Signature ______________________ **Signature Date** ______________________

Full Name (Parent/Guardian) ______________________ **Relationship to child** ______________________

Please complete and return this form by <<(DD/MM/YYYY)>>
